# Supplementary material for: Near-Infrared Genetically Encoded Positive Calcium Indicator Based on GAF-FP Bacterial Phytochrome
Source: Int J Mol Sci. 2019 Jul 16;20(14):3488. doi: 10.3390/ijms20143488 (PMC6678319; doi:10.3390/ijms20143488)
Supplement: Supplementary file 1 [file ijms-20-03488-s001.zip › Supplementary Information/Supplementary information.pdf]

Supplementary Information

Near-infrared genetically encoded positive calcium indicator based on GAF-FP bacterial phytochrome

Oksana M. Subach, Natalia V. Barykina, Konstantin V. Anokhin, Kiryl D. Piatkevich, and Fedor V. Subach

Supplementary Figures and Tables

**Table S1**	**2**
**Table S2**	**3,4**
**Figure S1**	**5**
**Figure S2**	**6**
**Figure S3**	**7**
**Figure S4**	**8**
**Figure S5**	**9,10**
**Figure S6**	**11**
**Figure S8**	**12**
**Figure S9**	**13**
**Video S1**	**14**
**Video S2**	**15**
**Supplementary Results and Discussion**	**16**
**Supplementary references**	**17**

Table S1. ***In vitro*** **properties of GAF-CaMP calcium indicator compared to smURFP and GAF-FP proteins.**

**Properties**		**Proteins**			
		**GAF-CaMP**		**smURFP**[a]	**GAF-FP**[b]
		**apo**	**sat**		
**Absorbance maximum (nm)**		643 (382)	644 (384)	642*	637 (379)
**Excitation maximum (nm)**		645	643	642	635
**Emission maximum (nm)**		675	675	670	670
**Quantum yield (%)** [c]		6.0±0.4	8.2±0.8	18	7.3
**ε (mM^{-1} cm^{-1})** [d]		64±1	87±3	180	49.8
**Brightness vs EGFP (%)** [e]		11.4±0.8	21±3	96	11
**p*K*a**		3.0; 9.0	3.0; >10.0	3.3*	4.0; 7.8
**ΔF/F (%)**	**Purified protein**	52±15		NA	NA
	**Bacteria**	95±9			
	**HeLa cells**	3.7±2%[f]			
**K_d (nM)** [g]		15±2 [1.7±0.4]			
**Photobleaching half-time (s)** [h]		ND	169±32	377±55*	ND

[a] Data from ref. [1]. Data marked with an asterisk were determined in this paper. NA, not applicable. ND, not determined. [b] Data from ref. [2]. [c] QY was determined at pH 7.20. GAF-FP was used as reference standard. [d] Extinction coefficient was determined relative to the Soret band at 382 and 384 nm. [e] Brightness was calculated as a product of the quantum yield and extinction coefficient and normalized to the brightness of EGFP that has an extinction coefficient of 56000 M^{-1} cm^{-1} and quantum yield of 0.6 [3]. [f] The ΔF/F response of GAF-CaMP indicator in HeLa cells was determined for its fusion with sfGFP upon addition of 2.5 μM Ionomycin. GAF-CaMP indicator was non-fluorescent in HeLa cells itself. [g] Calcium titrations were performed in the absence of Mg^{2+} ions. Hill coefficient is shown in square brackets. [h] The power of light from mercury-xenon lamp before the objective lens was 2.23 mW/cm^2. The properties of the purified GAF-CaMP indicator were also characterized using two-photon-absorption fluorescence spectroscopic analysis [4].

Table S2. List of primers.

Primer	Primer sequence (5′-3′)
**GAF-BglII**	actagatctATGCGGGCAGGTCCATCC
**GAF1**	ATGCGGGCAGGTCCATCCATTGATCTGTCTGGAATGTTGGCACCCGCTCTTGAGAGAATTAGGACTGCCG
**GAF2-r**	TACATTGTTGAAAAAGCAAAACGGTGTCGTCACAAAGGGCCCGAAGGCTCCCGGCAGTCCTAATTCTCTC
**GAF3**	GCTTTTTCAACAATGTACCGGCTACGACCGAGTAATGGTATATAGATTTGACGTTCAAGGTCATGACCAG
**GAF4-r**	AGCGGTTACCCAGATAGCTCTCCAGTCCAGGCACGTGACACTCACTAAATACCTGGTCATGACCTTGAAC
**GAF5**	GCTATCTGGGTAACCGCTACCCTAGTTCAATGGTTCCACAAATGGCAAGGCAACTGTATTTGCGGCAACG
**GAF6-r**	TGGAGATGACAAGGGCTCATTGACCGTCCTTGGTAAGTCAAGTCTACCCTCATTCGAACTCGTTGCCGCAAATACAGTTG
**GAF7**	GAGCCCTTGTCATCTCCAGTATCTCAAGGATATGGGGGTTCGCGCCACATTGGTTGTAAGCCTGGTTGTA
**GAF8-r**	TGAATCTCGGAAGATAGTGGTGACACACCACCAGTCCCCAAAGTTTTCCACCTACAACCAGGCTTACAAC
**GAF9**	CACTATCTTCCGAGATTCATCCACTTTGAACTCAGGGCATTTTGCAAAAGACTTGCGGAAAGAATTGCTA
**GAF10-r**	AGACTCAAGGGCAGCTATTCTCGTAGCAATTCTTTCCGCAAG
**GAF-EcoRI-r**	gtagaattcAGACTCAAGGGCAGCTATTC
**GAF-CM-6**	CTGGGTAACCGCTACCCTNNSNNSGACCAACTGACTGAAGAGC
**GAF-CM-6r**	GCTCTTCAGTCAGTTGGTCSNNSNNAGGGTAGCGGTTACCCAG
**CM-GAF-6**	CTATAGGTCGGCTGAGCTCANNSNNSAGTTCAATGGTTCCACAAATG
**CM-GAF-6r**	CATTTGTGGAACCATTGAACTSNNSNNTGAGCTCAGCCGACCTATAG
**GAF-CM-7**	GCAAGGCAACTGTATTTGNNSNNSGACCAACTGACTGAAGAGC
**GAF-CM-7r**	GCTCTTCAGTCAGTTGGTCSNNSNNCAAATACAGTTGCCTTGC
**CM-GAF-7**	CTATAGGTCGGCTGAGCTCANNSNNSCGGCAACGAGTTCGAATG
**CM-GAF-7r**	CATTCGAACTCGTTGCCGSNNSNNTGAGCTCAGCCGACCTATAG
**GAF-CM-8**	CAATGAGCCCTTGTCATCTCNNSNNSGACCAACTGACTGAAGAGC
**GAF-CM-8r**	GCTCTTCAGTCAGTTGGTCSNNSNNGAGATGACAAGGGCTCATTG
**CM-GAF-8**	CTATAGGTCGGCTGAGCTCANNSNNSCAGTATCTCAAGGATATG
**CM-GAF-8r**	CATATCCTTGAGATACTGSNNSNNTGAGCTCAGCCGACCTATAG
**GAF-CM-9**	GGACGGTCAATGAGCCCTNNSNNSGACCAACTGACTGAAGAGC
**GAF-CM-9r**	GCTCTTCAGTCAGTTGGTCSNNSNNAGGGCTCATTGACCGTCC
**CM-GAF-9**	CTATAGGTCGGCTGAGCTCANNSNNSTGTCATCTCCAGTATCTC
**CM-GAF-9r**	GAGATACTGGAGATGACASNNSNNTGAGCTCAGCCGACCTATAG

**GAF-CM-10**	CATCTCCAGTATCTCAAGNNSNNSGACCAACTGACTGAAGAGC
**GAF-CM-10r**	GCTCTTCAGTCAGTTGGTCSNNSNNCTTGAGATACTGGAGATG
**CM-GAF-10**	CTATAGGTCGGCTGAGCTCANNSNNSGATATGGGGGTTCGCGCC
**CM-GAF-10r**	GGCGCGAACCCCCATATCSNNSNNTGAGCTCAGCCGACCTATAG
**Fw-BglII-GECO**	GCTCGAGATCTATGGTCGACTCATCACGTCG
**Rv-EcoRI-GECO**	GCTCGGAATTCCTACTTCGCTGTCATCATTTG
**NCaMP-EcoRI-HindIII-r**	GTCAAGCTTGAATTCCTACTTCGCTGTCATCATTTG
**mCherry-HindIII-r**	GATAAGCTTTTACTTATACAGCTCGTC
**GAF-EcoRI-r2**	GTAGAATTCTCAAGACTCAAGGGCAGCTATTC

```
          1        10        20        30        40        50        60
          |        |         |         |         |         |         |
Library   MRAGPSIDLSGMLAPALERIRTAGSLRALCDDTVLLFQQCTGYDRVMVYRFDVQGHDQVF
GAF-CaMP  MRAGPSIDLSGMLTPALERIRTAGSLRALCDDTVLLFQQCTGYDRVMVYRFDVHGHDQVF

                 70        80        90       100       110       120
                 |         |         |         |         |         |
Library   SECHVPGLESYLGNRYPSSMVPQMARQLYLRQRVRMRVDLTYQGRSMSPXXDQLTEEQIA
GAF-CaMP  SECHVPGLESYLGNRYPSSMVPQLARQLYLRQRVRMRVDVTYQGRSMSPLLDQLTEEQIA

                130       140       150       160       170       180
                 |         |         |         |         |         |
Library   EFKEAFSLFDKDGDGTITTKELGTVMRSLGQNPTEAELQDMINEVDADGDGTIDFPEFLT
GAF-CaMP  EFKEAFSLFDKDGDGTITTKELGTVMRSLGQNPTEAELRVMINEVDADGDGTIDFPEFLT
                   * * * * *  *                        * * * * *  *

                190       200       210       220       230       240
                 |         |         |         |         |         |
Library   MMARKMKYRDTEEEIREAFGVFDKDGNGYISAAELRHVMTNLGEKLTDEEVDEMIREADI
GAF-CaMP  MMARKMKYRDTEEEIREAFGVFDKDGNGYISAAELRHVMTNLGEKLTDEEVGEMIREADI
                                * * * * *  *                        *

                250       260       270       280       290       300
                 |         |         |         |         |         |
Library   DGDGQVNYEEFVQMMTAKGGSSSRRKWNKTGHAVRAIGRLSSXXCHLQYLKDMGVRATLV
GAF-CaMP  DGDGQVNYEEFVQMMIAKGGSSSRRKWNKTGHAVRAIGRLSSGGCHLRYLKDMGVRASLV
          * *   *  *

                310       320       330       340
                 |         |         |         |
Library   VSLVVGGKLWGLVVCHHYLPRFIHFELRAFCKRLAERIATRIAALES
GAF-CaMP  VSLVVGGKLWGLVVCYHYLPRYIHYELRAFCKRLAERIAARIAALES
```

Figure S1. Alignment of the amino acid sequences for the original library L9 and GAF-CaMP calcium indicator. Alignment numbering follows that of original library L9. Mutations in GAF-CaMP related to the initial library L9 including linkers between fluorescent and indicator parts are highlighted in yellow. The residues which are suggested within 4.5, 4.5-5.5 and 5.5-6.5 Å surrounding to BV chromophore according to the X-ray structure of PaBphP (3C2W) are highlighted in grey, cyan and red colors, respectively. Residues in CaM-part that assumed to bind Ca^{2+} ions are selected with stars. M13-peptide is underlined.

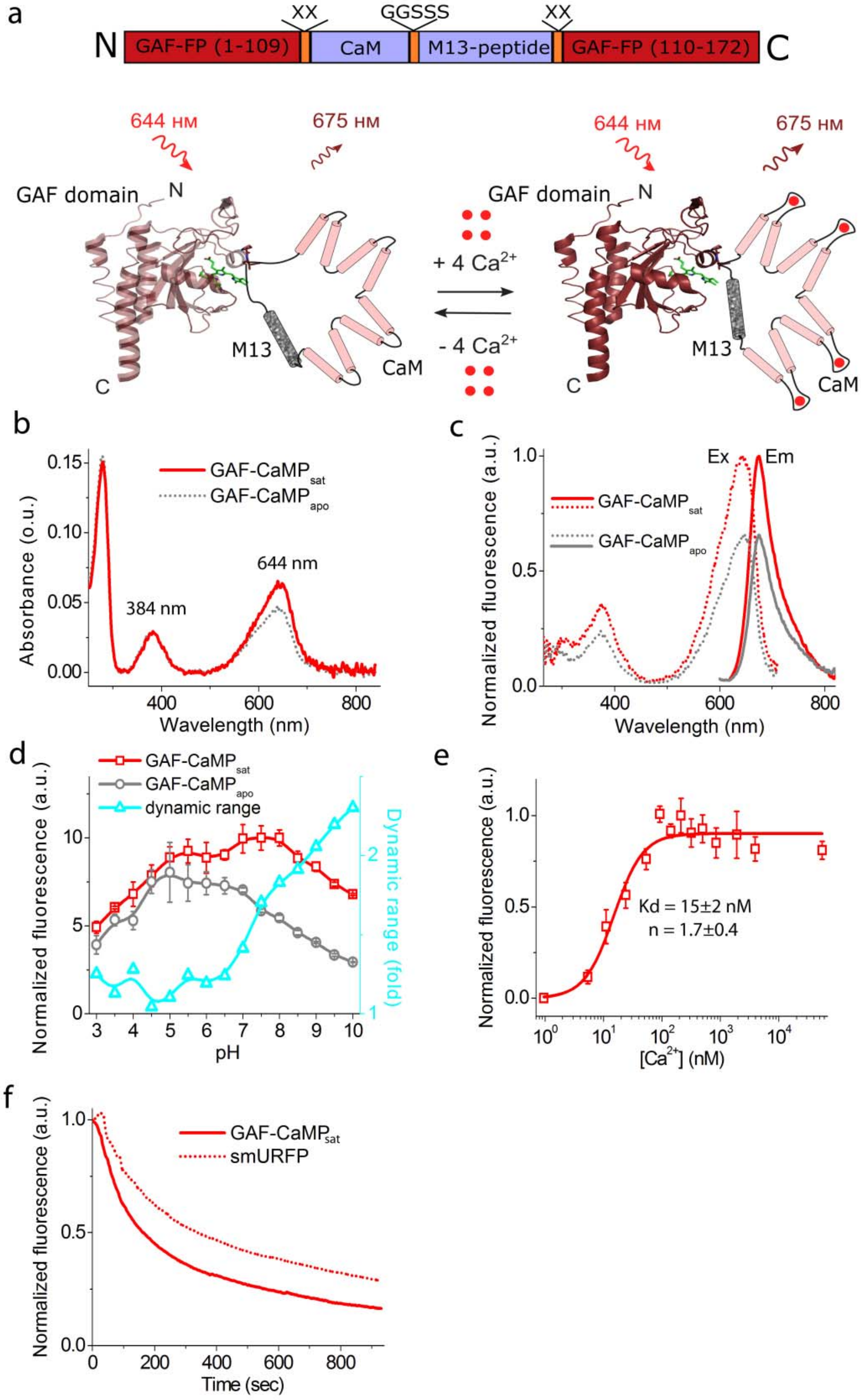

Figure S2. ***In vitro*** **properties of the purified GAF-CaMP indicator. (a)** A scheme of the original library for optimization of linkers in the GAF-CaMP indicator and suggested mechanism of its function based on crystal structure of GAF domain (pdb 3C2W). **(b)** Absorbance spectra for GAF-CaMP in Ca^{2+}-bound and Ca^{2+}-free states at pH 7.2. **(c)** Excitation and emission spectra for GAF-CaMP in Ca^{2+}-bound and Ca^{2+}-free states, pH 7.2. **(d)** Fluorescence intensity for GAF-CaMP in Ca^{2+}-bound and Ca^{2+}-free states as a function of pH. Three replicates were averaged for analysis. Error bars represent the standard deviation. **(e)** Ca^{2+} titration curves for GAF-CaMP in the presence of 1 mM $MgCl_2$, pH 7.2. Three replicates were averaged for analysis. Error bars represent the standard deviation. **(f)** Photobleaching curves for GAF-CaMP in Ca^{2+}-bound state and smURFP. The power of light before objective lens was 2.23 mW/cm^2. Nine to fourteen replicates were averaged for analysis.

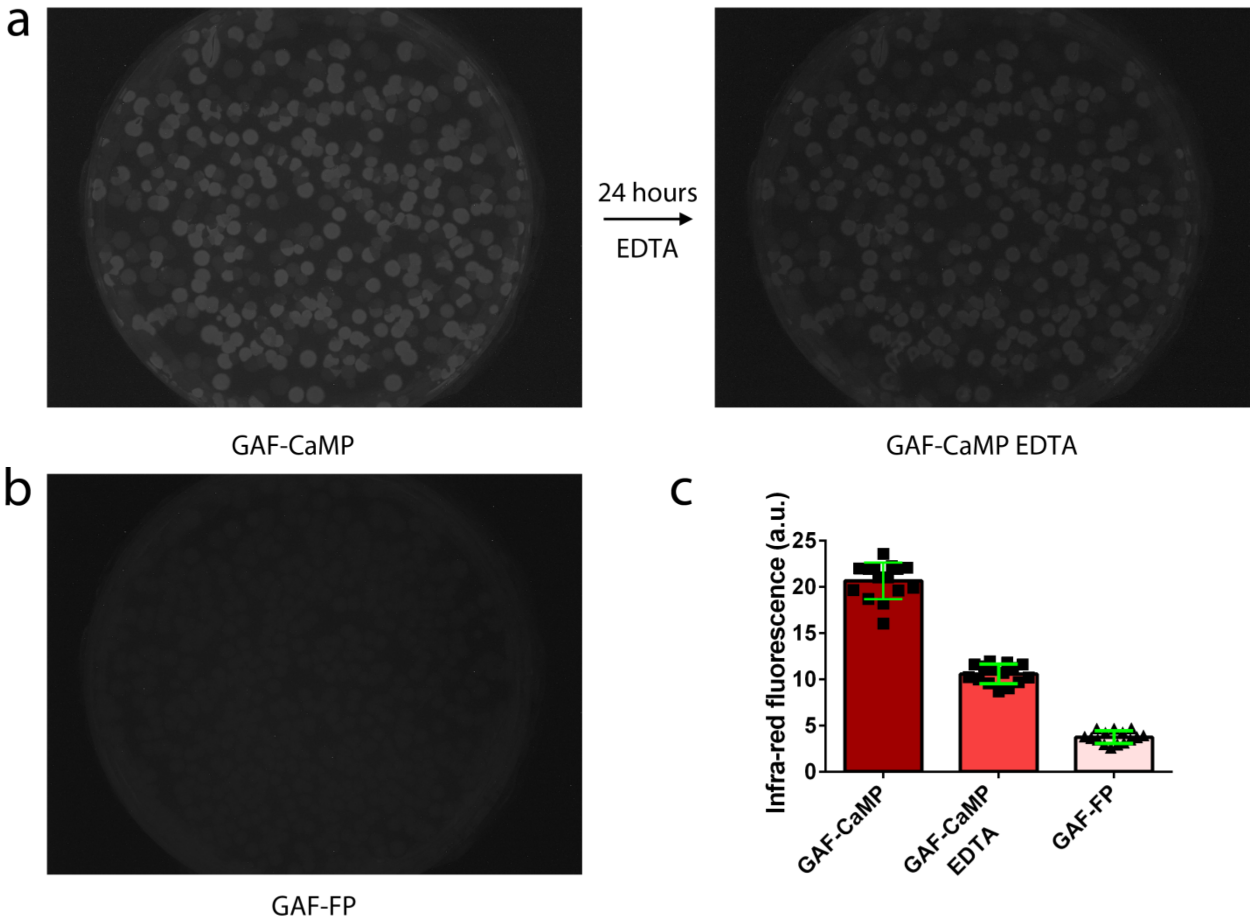

Figure S3. Brightness and response of the GAF-CaMP indicator to Ca^{2+} variations in bacterial cells. Fluorescent images of BW25113/pWA23 bacterial cells expressing near-infrared GAF-CaMP calcium indicator before and 24 hours after spraying of 100 mM EDTA **(a)** or GAF-FP fluorescent protein **(b)**. For the panels a and b imaging conditions were the same. **(c)** The bars illustrate averaged near-infrared fluorescence of bacterial colonies expressing the GAF-CaMP indicator, GAF-CaMP after spraying of EDTA and parental GAF-FP protein. Each dot represents fluorescence value from one of fifteen colonies. Error bars represent the standard deviation for the average fluorescence across 15 colonies.

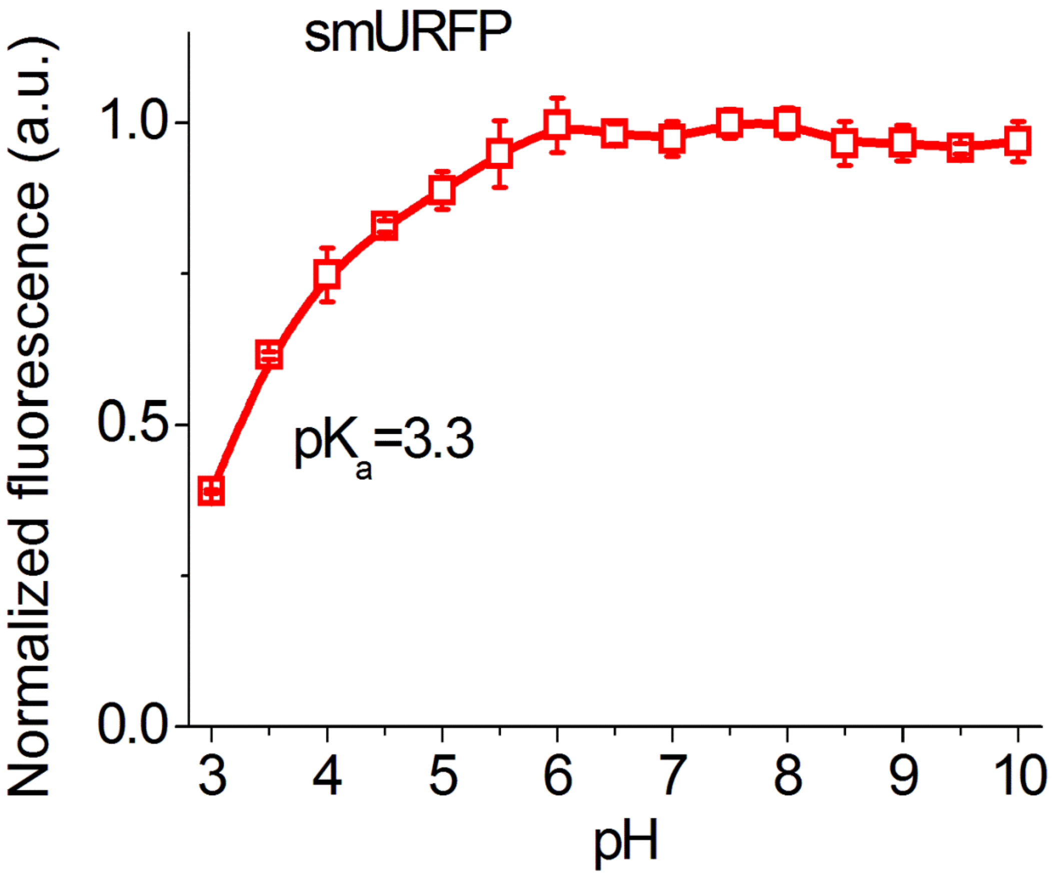

Figure S4. pH dependence of smURFP fluorescence. Average of three repeats and SD are shown.

NES-GAF-CaMP2-sfGFP-stop gene:

ATGCTTCAACTTCCTCCTCTTGAACGTCTTACTCTTTCGAGATCTATGCGGGCAGGTCCATCCATTG
ATCTGTCTGGAATGTTGACGCCCGCTCTTGAGAGGATTAGGACTGCCGAGAGCCTTCGGGCCCTC
TGTGACGACACCGTTTTGCTTTTTCGACAATGTACCGGCTACGACCGTGTAATGGTGTACAGATTT
GACGTTCATGGTCAAGACCAGGTATTTAGTGAGTGTCATGCGCCGGGACTGGAGAGCTATCTGGG
TAACCGCTACCCTAGCTCAATGGTTCCACAATTGGCAAGGCAACTGTATCTGCGGCAACGAGTTC
GAATGAGGGCGGACGTGGCTTACCGAGGGCGGCCAACGAGCCCTTTGCTGGACCAACTGACTGA
AGAGCAGATCGCAGAATTTAAAGAGGCTTTCTCCCTATTTGACAAGGACGGGGATGGGACAGTA
ACCACCAAGGAGCTGGGGACGGTGATGCGGTCTCTGGGGCAGAACCCCACAGAAGCAGAGCTG
CGGGTCATGATCAATGAAGTAGATGCCGACGGTGACGGCACACTCGACTACCCTGAGTTCCTGA
CAATGATGGCAAGAAAAATGAAATACAGGGACACGGAAGAAGAAATTAGAGAAGCGTTCGGTG
TGTTTGATAAGGATGGCAATGGCTACATCAGTGCAGCAGAGCTTCGCCATGTGTTGACAAACCTT
GGAGAGAAGTTAACAGATGAAGAGGTTGGTGAATTGATCAGGGAAGCAGCCATCGATGGGGAT
GGTCAGGTGAACTACGAGGAGTTCGTACAAATGATGACAGCGAAGGGCGGTTCAGGAGGCGGG
TGCTCATCGCGTCGTATGTGGAATAAGGCAGGTCACGCAGTCAGAGCTATAGGTCGGCTGAGCTC
TGGTGGTTGTCATCTCCGGTACCTCAAAGATATGGGGGTCCGCGCCTCATTGGTTGTAAGCCTGGT
TGTAGGCGGAAAACTTTGGGGACTGGTGGTGTGTTACCACTATCTTCCGAGATACATCCACTATG
AACTCAGGGCATTTTGCAGAAGACTTGCGGATGGAATTGCAGCGAGAATAGCTGCCCTTGAGTCT
GAATTCATGGTGAGCAAGGGCGAGGAGCTGTTCACCGGGGTGGTGCCCATCCTGGTCGAGCTGG
ACGGCGACGTAAACGGCCACAAGTTCAGCGTGCGCGGCGAGGGCGAGGGCGATGCCACCAACG
GCAAGCTGACCCTGAAGTTCATCTGCACCACCGGCAAGCTGCCCGTGCCCTGGCCCACCCTCGTG
ACCACCCTGACCTACGGCGTGCAGTGCTTCAGCCGCTACCCCGACCACATGAAGCGCCACGACT
TCTTCAAGTCCGCCATGCCCGAAGGCTACGTCCAGGAGCGCACCATCAGCTTCAAGGACGACGG
CACCTACAAGACCCGCGCCGAGGTGAAGTTCGAGGGCGACACCCTGGTGAACCGCATCGAGCTG
AAGGGCATCGACTTCAAGGAGGACGGCAACATCCTGGGGCACAAGCTGGAGTACAACTTCAAC
AGCCACAACGTCTATATCACCGCCGACAAGCAGAAGAACGGCATCAAGGCCAACTTCAAGATC
CGCCACAACGTGGAGGACGGCAGCGTGCAGCTCGCCGACCACTACCAGCAGAACACCCCCATC
GGCGACGGCCCCGTGCTGCTGCCCGACAACCACTACCTGAGCACCCAGTCCGTGCTGAGCAAAG
ACCCCAACGAGAAGCGCGATCACATGGTCCTGCTGGAGTTCGTGACCGCCGCCGGGATCACTCA
CGGCATGGACGAGCTGTATAAGTAA

IMS-R-GECO1-stop gene:

atggcggctctgagaagttgggtgactcggagcgtctgctctctgttcaggtacagacagcgttttcctgtcttggctaactctaagaaacgctgtttctcag
aattgataaaaccatggcacaaaactgtgttgactggatttggcatgacactgtgtggatcaggaggatcaagatctatggtcgactcatcacgtcgtaagt
ggaataaggcaggtcacgcagtcagagctataggtcggctgagctcacccgtggtttccgagcggatgtaccccgaggacggcgccctgaagagcga
gatcaagaaggggctgaggctgaaggacggcggccactacgccgccgaggtcaagaccacctacaaggccaagaagcccgtgcagctgcccggcgc
ctacatcgtcgacatcaagttggacatcgtgtcccacaacgaggactacaccatcgtggaacagtgcgaacgcgccgagggccgccactccaccggcgg
catggacgagctgtacaagggaggtacaggcgggagtctggtgagcaagggcgaggaggataacatggccatcatcaaggagttcatgcgcttcaag
gtgcacatggagggctccgtgaacggccacgagttcgagatcgagggcgagggcgagggccgcccctacgaggcctttcagaccgctaagctgaag
gtgaccaagggtggcccccctgcccttcgcctgggacatcctgtcccctcagttcatgtacggctccaaggcctacattaagcacccagccgacatccccgac
tacttcaagctgtccttccccgagggcttcaggtgggagcgcgtgatgaacttcgaggacggcggcattattcacgttaaccaggactcctccctgcagga
cggcgtattcatctacaaggtgaagctgcgcggcaccaacttcccccccgacggccccgtaatgcagaagaagaccatgggctgggaggctacgcgtg
accaactgactgaagagcagatcgcagaatttaaagaggctttctccctatttgacaaggacggggatgggacgataacaaccaaggagctggggacg
gtgatgcggtctctggggcagaaccccacagaagcagagctgcaggacatgatcaatgaagtagatgccgacggtgacggcacattcgacttccctga
gttcctgacgatgatggcaagaaaaatgaatgacacagacagtgaagaggaaattagagaagcgttccgcgtgtttgataaggacggcaatggctaca

tcggcgcagcagagcttcgccacgtgatgacagaccttggagagaagttaacagatgaggaggttgatgaaatgatcagggtagcagacatcgatgg
ggatggtcaggtaaactacgaagagtttgtacaaatgatgacagcgaagtag

H2B-B-GECO1-stop gene:

atgccagagccagcgaagtctgctcccgccccgaaaaagggctccaagaaggcggtgactaaggcgcagaagaaaggcggcaagaagcgcaagcg
cagccgcaaggagagctattccatctatgtgtacaaggttctgaagcaggtccaccctgacaccggcatttcgtccaaggccatgggcatcatgaattcgtt
tgtgaacgacattttcgagcgcatcgcaggtgaggcttcccgcctggcgcattacaacaagcgctcgaccatcacctccagggagatccagacggccgtg
cgcctgctgctgcctggggagttggccaagcacgccgtgtccgagggtactaaggccatcaccaagtacaccagcgctaagagatctatggtcgactcac
cacgtcgtaagtggaataagacaggtcacgcagtcagagctataggtcggctgagctcaccagagaacgtctatatcaaggccgacaagcagaagaac
ggcatcaaggcgaacttcaagatccgccacaacatcgagggcggcggcgtgcagctcgcctaccactaccagcagaacacccccatcggcgacggccc
cgtgctgctgcccgacaaccactacctgagcgtgcagtccatactttcgaaagaccccaacgagaagcgcgatcacatggtcctgctggagttcgtgacc
gccgccgggatcactctcggcatggacgagctgtacaagggcggtaccggagggagcgaatccatggtgagcaagggcgaggagctgttcaccggg
gtggtgcccatccaggtcgagctggacggcgacgtaaacggccacaagttcagcgtgtccggcgagggtgagggcgatgccacctacggcaagctga
ccctgaagttcatctgcaccaccggcaagctgcccgtgccctggcccaccctcgtgaccaccctgtcacacggcgtgcagtgcttcagccgctaccccgacc
acatgaagcagcacgacttcttcaagtccgccatgcccggaggctacatccaggagcgcaccatcttcttcaaggacgacggcaactacaagacccgcgc
cgaggtgaagttcgagggcgacaccctggtgaaccgcatcgagctgaagggcatcgacttcaaggaggacggcaacatcctggggcacaagctgga
gtacaacacgcgtggccaactgactgaagagcagatcgcagaatttaaagaggctttctccctatttgacaaggacggggatgggacgataacaaccaa
ggagctggggacggtgatgcggtctctggggcagaaccccacagaagcagagctgcaggacatgatcaatgaagtagatgccgacggtgacggcac
aatcgacttccctgagttcctgacaatgatggcacctaaaatgcaggacacagacagtgaagaagaaattagagaagcgttccgtgtgtttgataaggac
ggcaatggctacatcggcgcagcagagcttcgccacgtgatgacaaaccttggagagaagttaacagatgaagaggttgatgaaatgatcagggaagc
agacatcgatggggatggtcaggtaaactacgaagagtttgtacaaatgatgacagcgaagtag

Figure S5. Nucleotide sequences of NES-GAF-CaMP2-sfGFP, IMS-R-GECO1 and H2B-B-GECO1 proteins.

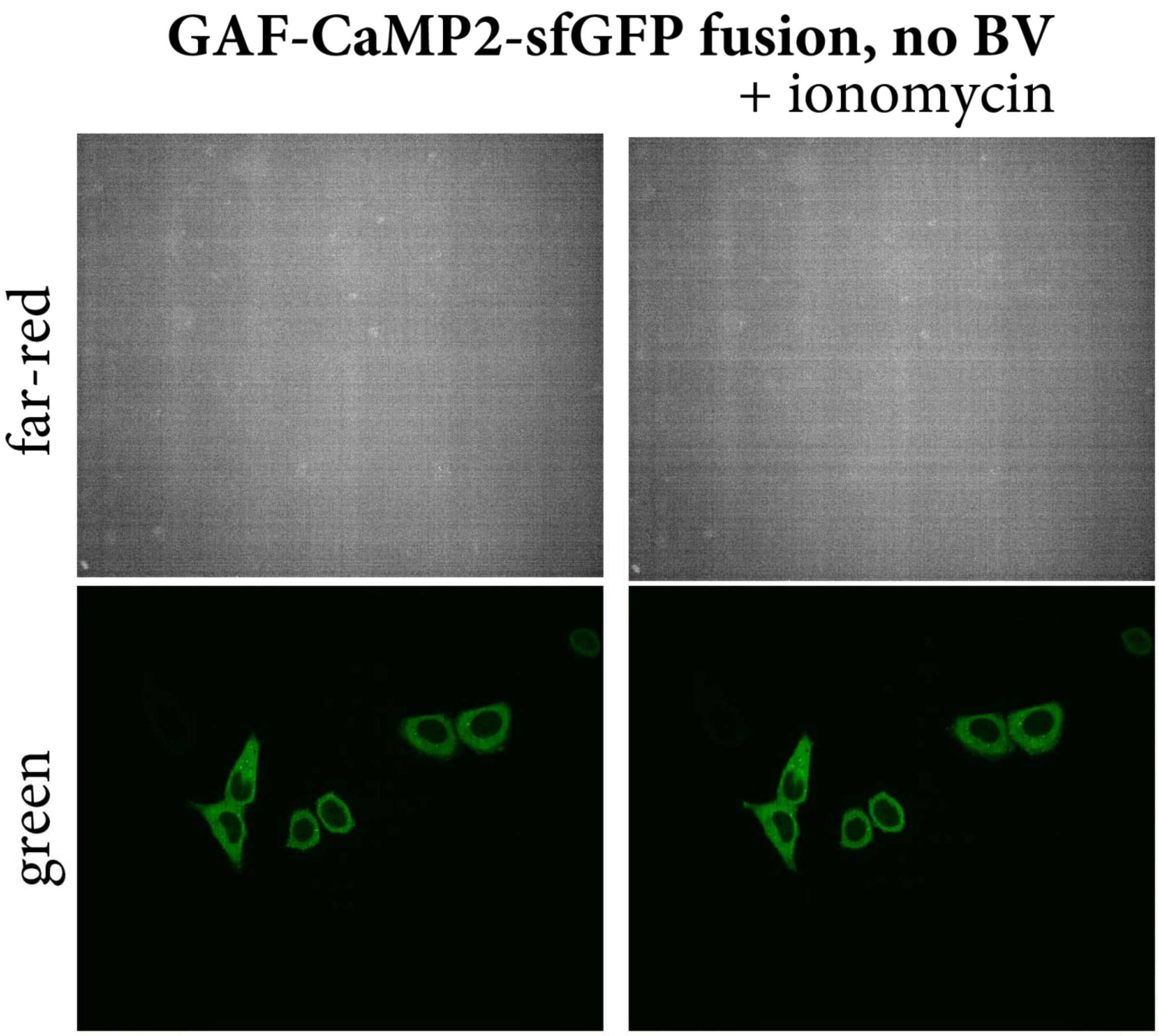

Figure S6. Optimization of GAF-CaMP2 expression in mammalian cells. Confocal images of HeLa Kyoto cells expressing green/far-red NES-GAF-CaMP2-sfGFP fusion before and after addition of the 2.5 μM ionomycin. No BV was supplied to the cell culture. Green (ex. 488 nm, em. 525/50 nm) and far-red (ex. 640 nm, em. 685/40 nm) channels are shown.

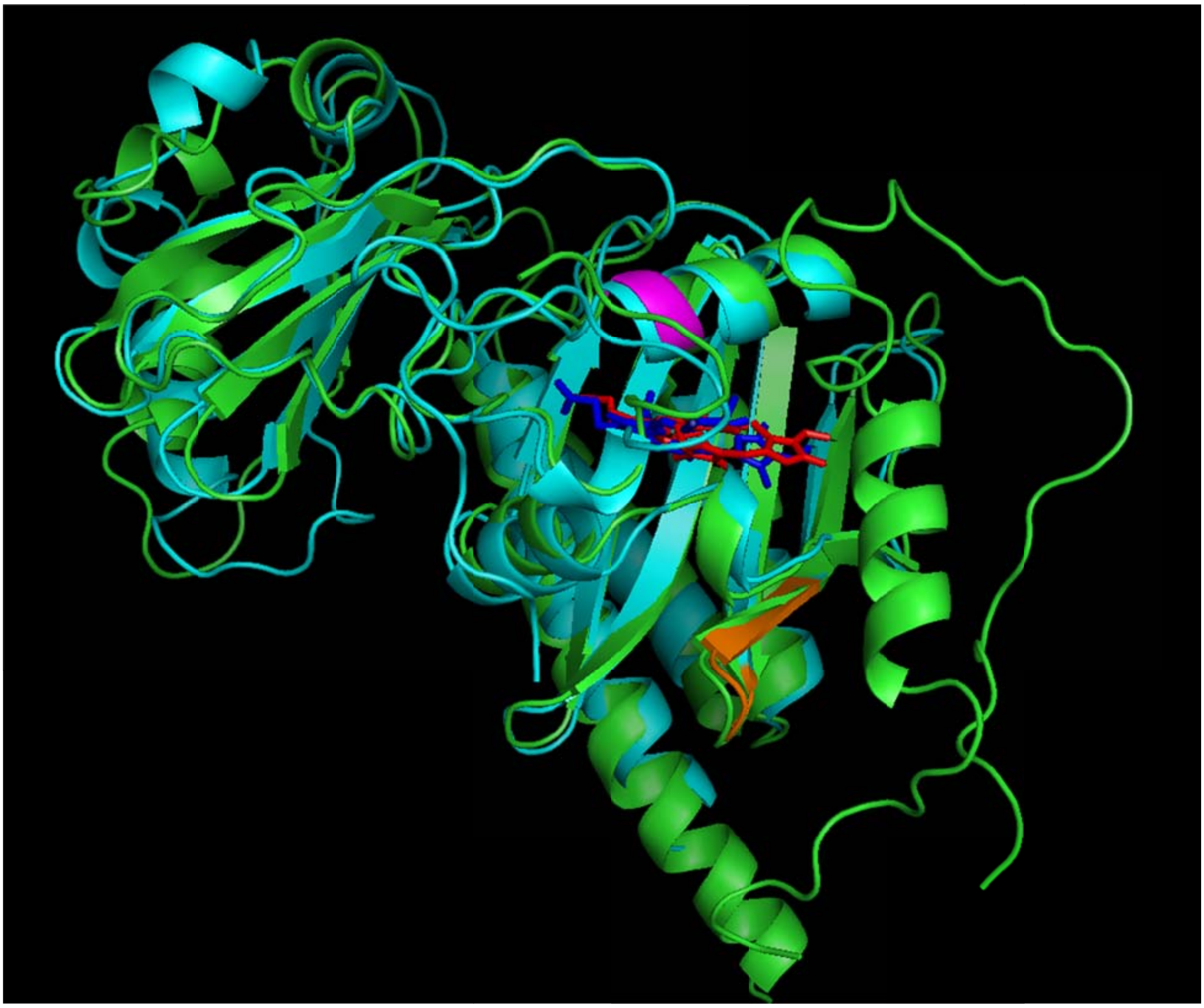

Figure S7. Comparison of location of calcium binding domains insertion sites for GAF-CaMP2 and NIR-GECO1 indicators. X-ray structures of *Pa*BphP (PDB 3C2W in green; BV chromophore in red) and *Dr*BphP (PDB 2O9B in cyan; BV chromophore in blue) bacteriophytochromes were aligned in Swiss-PdbViewer 4.0.4 and represented in PyMol 0.99. The insertion sites of calcium binding domains are highlighted in pink (GAF-CaMP2, residues PI) and orange (NIR-GECO1, residues APDATGE) colors.

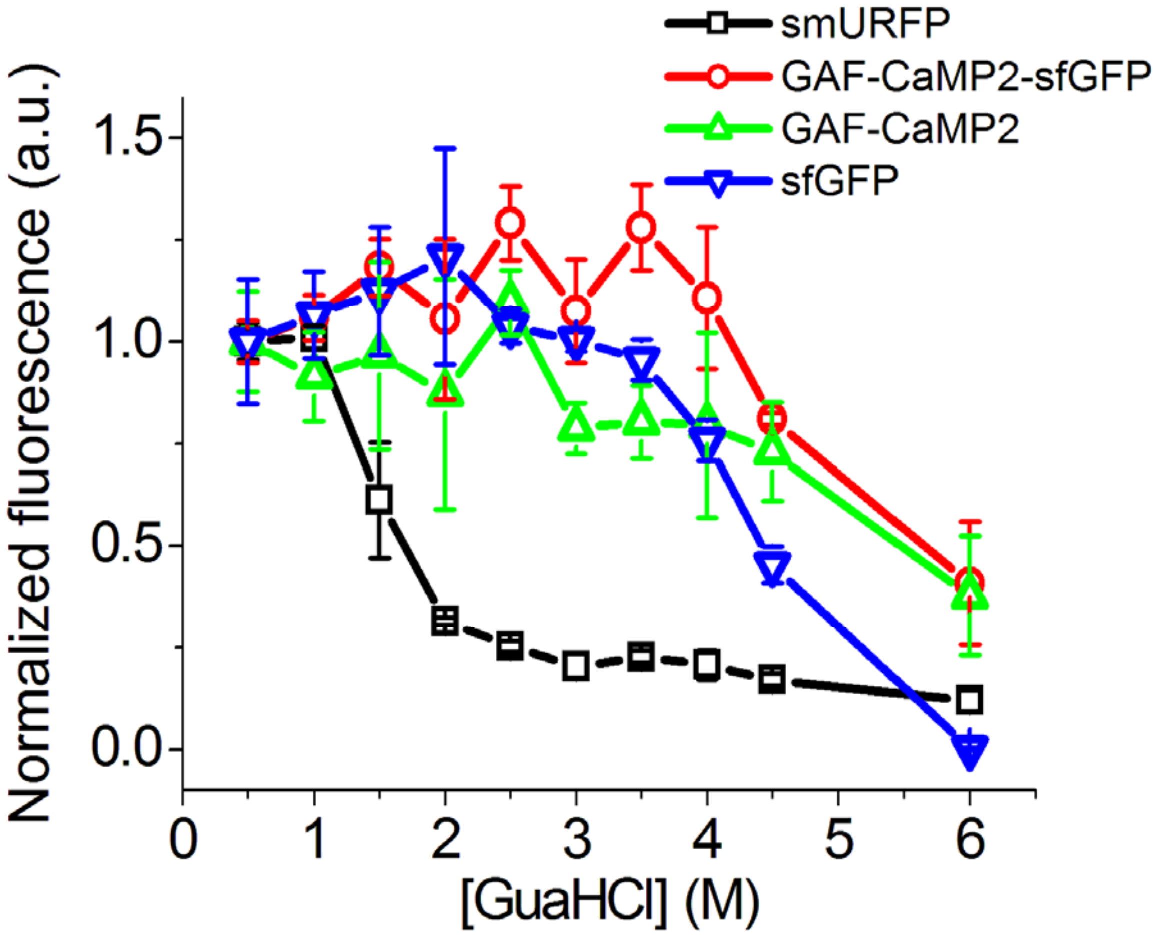

Figure S8. Comparison of in vitro stabilities for NIR FPs. The fluorescence of NIR FPs was measured after 20 h of incubation with different concentrations of guanidine hydrochloride (GuHCl) denaturant. The data were normalized to the fluorescence of NIR FPs in 0.5 M GuHCl buffered solution (averaged across 3 repeats). Calculated concentrations of guanidine hydrochloride, in which 50% fluorescence was retained, were 1.7, 5.5, and 5.7 M for smURFP, GAF-CaMP2 and GAF-CaMP2-sfGFP proteins, respectively.

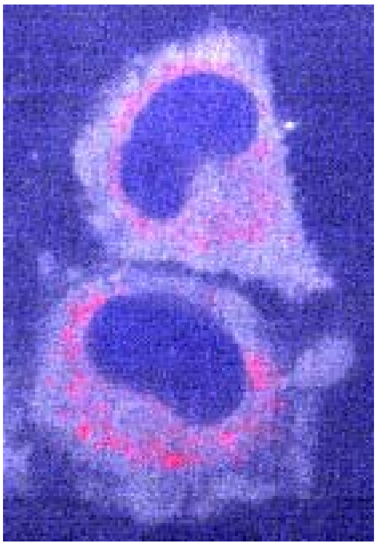

Video S1. Visualization of ionomycin-induced calcium transients in three organelles of mammalian cells using GAF-CaMP2 indicator and four-color fluorescence confocal microscopy. Single frame is for the time of 4 min 09 sec is shown. This field of view corresponds to the Figure 5a shown in the main text.

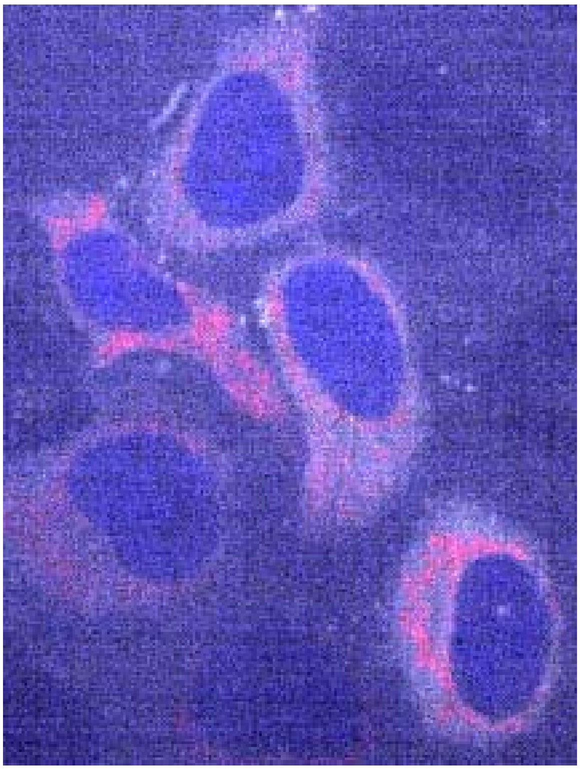

Video S2. Visualization of thapsigargin-induced calcium transients in three organelles of mammalian cells using GAF-CaMP2 indicator and four-color fluorescence confocal microscopy. Single frame is for the time of 4 min 44 sec is shown. This field of view corresponds to the Figure 5b shown in the main text.

Supplementary Results and Discussion

Calcium-dependent response and brightness of the GAF-CaMP indicator in bacterial cells

To characterize the behavior of the GAF-CaMP indicator in cells, we investigated its brightness and response to the Ca^{2+} transients in the BW25113/pWA23 live bacterial cells. Under the same conditions in bacterial cells, the GAF-CaMP indicator demonstrated an average brightness that was 5.5-fold higher than for the GAF-FP protein (ESI Figure S3c). After the administrating 100 mM EDTA solution to Petri dishes with colonies expressing the GAF-CaMP indicator, it demonstrated an averaged maximal ΔF/F value of 95±9% in approximately 24 hours (ESI Figure S3a, c). This contrast was 1.8-fold higher as compared with contrast on purified protein, probably, because of protein oxidation, denaturation or degradation during purification procedure or other reasons. Thus, in bacterial cells, the GAF-CaMP indicator demonstrated a 95% fluorescence ΔF/F response to variations in the Ca^{2+} concentration and was 5.5-fold brighter than GAF-FP progenitor.

Overall, the results suggested that in terms of brightness, ΔF/F response and calcium sensitivity, the GAF-CaMP indicator was appropriate for monitoring of calcium transients in bacterial cells.

Supplementary references

1. Rodriguez, E. A.; Tran, G. N.; Gross, L. A.; Crisp, J. L.; Shu, X.; Lin, J. Y.; Tsien, R. Y., A far-red fluorescent protein evolved from a cyanobacterial phycobiliprotein. *Nature methods* **2016,** 13, (9), 763-9.
2. Rumyantsev, K. A.; Shcherbakova, D. M.; Zakharova, N. I.; Emelyanov, A. V.; Turoverov, K. K.; Verkhusha, V. V., Minimal domain of bacterial phytochrome required for chromophore binding and fluorescence. *Sci Rep* **2015,** 5, 18348.
3. Heim, R.; Cubitt, A. B.; Tsien, R. Y., Improved green fluorescence. *Nature* **1995,** 373, (6516), 663-4.
4. Lanin, A. A.; Chebotarev, A. S.; Barykina, N. V.; Subach, F. V.; Zheltikov, A. M., The whither of bacteriophytochrome-based near-infrared fluorescent proteins: Insights from two-photon absorption spectroscopy. *Journal of biophotonics* **2019,** 12, (5), e201800353.
